# Predicting Active Users' Personality Based on Micro-Blogging Behaviors

**DOI:** 10.1371/journal.pone.0084997

**Published:** 2014-01-22

**Authors:** Lin Li, Ang Li, Bibo Hao, Zengda Guan, Tingshao Zhu

**Affiliations:** 1 Institute of Psychology, Chinese Academy of Sciences, Beijing, China; 2 School of Computer and Control, University of Chinese Academy of Sciences, Beijing, China; University of Illinois at Chicago, United States of America

## Abstract

Because of its richness and availability, micro-blogging has become an ideal platform for conducting psychological research. In this paper, we proposed to predict active users' personality traits through micro-blogging behaviors. 547 Chinese active users of micro-blogging participated in this study. Their personality traits were measured by the Big Five Inventory, and digital records of micro-blogging behaviors were collected via web crawlers. After extracting 845 micro-blogging behavioral features, we first trained classification models utilizing Support Vector Machine (SVM), differentiating participants with high and low scores on each dimension of the Big Five Inventory. The classification accuracy ranged from 84% to 92%. We also built regression models utilizing PaceRegression methods, predicting participants' scores on each dimension of the Big Five Inventory. The Pearson correlation coefficients between predicted scores and actual scores ranged from 0.48 to 0.54. Results indicated that active users' personality traits could be predicted by micro-blogging behaviors.

## Introduction

Personality refers to “the consistent behavior patterns and interpersonal processes originating within the individual” [1]. From the perspective of psychology, personality is an essential mental function for distinguishing one person from the others [2]. Because of its relative consistency over ages and prediction of consequential outcomes (e.g., psychological well-being, mental health, physical health, quality of intimate relationship, career choice and satisfaction, performance in workplace and political ideology), personality has been regarded as one of the most important topics in psychological research [3].

Since personality is an implicit psychological construct which cannot be observed directly, it has to be measured through valid and explicit behavioral indicators (i.e., behavioral samples) [4]. That is, outcome of personality testing depends on how personality-related behavioral indicators are measured. Currently, quite a few methods for measuring personality have been developed [5]. Among them, self-report technique is the most widely-used method [6]. However, because of its limitations in participant recruitment and resource consumption, self-report method needs to be improved [7], [8].

The emergence of micro-blogging service has given us an opportunity to change traditional psychological testing. Nowadays, micro-blogging has become one of the most popular web services around the world. For example, Sina Weibo (http://weibo.com/), which is a leading micro-blogging service in China, has over 300 million users. In term of features, Sina Weibo is similar to Twitter, the most popular America platform for micro-blogging. Registered Sina Weibo users are allowed to post with the 140 character limit, insert emoticons and multi-media documents (e.g., image, music and video files) into micro-blogs, interact with other users (e.g., leaving messages, forwarding of micro-blogs and posting or re-posting comments) using @ mentions, follow other users to make their micro-blogs available at users' own timeline, and add selected micro-blogs to my favorites. Through micro-blogging, users are motivated to make greater self-disclosure and self-presentation [9]. In addition to that, they are free to express opinions and views concerning their perception and concerns [10]. Because of its richness and availability, micro-blogging has been regarded as an ideal web-based platform to examine human psychological profiles and differences.

In order to deeply understand web users, psychologists are interested in the relationship between psychological features (e.g., personality traits) and web use behaviors. Previous studies have demonstrated that personality is a determining factor toward web use behaviors [11], [12]. That is, web users' personality traits could be manifested on web use behaviors [13], [14]. Subsequent studies have repeatedly confirmed this finding on various kinds of web-based media or applications (e.g., SNS) [15], [16], [17]. For blogging or micro-blogging, Qiu et al found that extraverts prefer to use micro-blogging for relieving their existential anxiety [18]; Nowson and Oberlander suggested to automatically identify users' personality traits through blog texts utilizing a classification algorithm [19]; Park, Kim and Kim demonstrated that the layout of graphic design elements in the second generation blog could be used to create the impression of a cyber-personality [20].

In summary, previous studies mostly focus on correlations between personality traits and web use behaviors. Such correlation-based conclusions could not be used directly to predict web users' personality traits, and measures of web use behaviors rely on self-report technique. Because of the high flexibility, interactivity and complexity of web use behaviors, self-report technique might lead to conflicting measuring results [21], [22].

Recently, a few researchers tend to predict web users' personality traits through their digital records of web use behaviors. Globeck intended to predict web users' personality traits through text features on Facebook and Twitter [23], [24]. Quercia proposed to predict web users' personality traits through three features (i.e., following, followers and listed counts) available on profiles of Twitter [25]. However, these studies were limited by small sizes and sampling techniques. The performance of established prediction models were evaluated by goodness-of-fit indices (e.g., Mean Absolute Error or Root Mean-Square Error), which might be sensitive to sampling biases. Thus, to confirm previous results, there is a need to provide further evidence from a new approach.

This study aimed to examine the relationship between personality traits and digital records of micro-blogging behaviors based on over 500 samples, which were derived from a total of 1,953,485 active users. Differing from previous studies, we only focus on active users, whose digital records of micro-blogging behaviors might be rich enough for further analyses. It was hypothesized that active users' personality traits could be predicted by their micro-blogging behaviors.

## Method

### Participants

A total of 547 Chinese Sina Weibo users agreed to take part in this study voluntarily (214 men and 333 women and 23.66±5.28 years old on average). All participants were over 18 years.

### Ethics Statement

This study was conducted via the Web, and participants were recruited from different regions in China. Thus, we used an electronic informed-consent form instead of a written one. Specifically, before entering this study, participants were presented with an electronic informed-consent form on a web page. At the bottom of this page, two buttons were provided: “I agree” and “I disagree”. If one clicks “I agree”, he/she would be treated as a regular participant with an informed consent agreement; if one clicks “I disagree”, he/she would not be selected as a participant. The experimental design was approved by the Institutional Review Board of the Institute of Psychology, Chinese Academy of Sciences.

### Measurement

#### Personality Traits

In the field of personality psychology, the Big-Five personality structure is the most widely accepted theoretical framework [1], [26]. Quite a few measuring instruments have been developed to assess the Big-Five personality traits, such as the NEO by Costa and McCrae [27] and the TDA by Goldberg [28]. Among them, John's 44-item Big Five Inventory (BFI) is one of the most widely used brief measures of the Big-Five personality traits [29]. The instrument has five subscales, and each measures a Big-Five dimension labeled Extraversion (8 items), Agreeableness (9 items), Conscientiousness (9 items), Neuroticism (8 items), and Openness (10 items). The items consist of short and easy-to-understand phrases to assess the prototypical traits defining each of the Big-Five dimensions. An example item that belongs to the Extraversion subscale is “I am someone who is talkative”. Participants rated themselves on each item by a 5-point Likert-type scale (1 =  strongly disagree to 5 =  strongly agree). The Chinese version of BFI with satisfying psychometric properties is freely available at John's personal web page (http://www.ocf.berkeley.edu/~johnlab/bfi.php). In this study, participants were instructed to complete the Chinese version of BFI via Internet. The Cronbach's alphas for the Extraversion, Agreeableness, Conscientiousness, Neuroticism and Openness subscales were 0.73, 0.67, 0.74, 0.71 and 0.72, respectively, in our data. For all 547 participants, both mean value and standard deviation of scores on each personality dimension were estimated (see **Table 1**).

**Table 1:** Mean Value and Standard Deviation of Scores on Five Dimensions of the Big-Five Personality Traits (*n* = 547).

	Extraversion	Agreeableness	Conscientiousness	Neuroticism	Openness
Mean Value	3.20	3.65	3.15	3.08	3.61
Standard Deviation	0.66	0.56	0.60	0.65	0.56

Notes. Each dimension of personality is scored from 1.00 to 5.00.

#### Micro-Blogging Behaviors

We collected digital records of users' micro-blogging behaviors by calling Application Programming Interfaces (APIs) provided by Sina Weibo. With users' permission, we could download their digital records through APIs shown in **Table 2**. After extracting behavioral features from downloaded data, we selected more effective features for predicting personality traits.

**Table 2:** Details of APIs Provided by Sina Weibo.

Categories	Description
Users/show	detailed information of a user's profile
Blog/user_timeline	list of a user's micro-blogs updates
Trends	list of a user's trending topics selection
Tag	list of a user's tags selection
Friendships/friends	detailed information of friends whom a user follows
Friendships/friends/ids	list of registration IDs of friends whom a user follows
Friendships/followers/ids	list of registration IDs of a user's followers
Friendships/friends/bilateral	detailed information of a user's mutual friends

### Research Procedure

A three-step procedure was executed in this study: (a) constructing sampling pool, (b) selecting active users and (c) collecting data.

#### Constructing Sampling Pool

Currently, Sina Weibo has over 300 million registered users, producing more than 100 million micro-blogs daily [30]. To select active users from a huge number of Sina Weibo users for further analyses, it is necessary to construct an appropriate sampling pool. Specifically, in this study, by means of breadth-first search, one randomly selected user who followed ten friends was selected as a seed user to initiate crawling the social network of Sina Weibo users. After acquiring 15,767,158 users, the social network began to grow extremely slowly. We stopped the crawling, and randomly selected 291,039 users from 15,767,158 users as expanded seed users, who have 1000–3300 followers. Through friend connections of expanded seed users, we could resume crawling the remaining social network. Then, 1,116,408,085 connections were obtained. Finally, a total of 99,925,821 Sina Weibo users remained in the sampling pool after duplicate names were removed.

#### Selecting Active Users

It is expected that active users' digital records of micro-blogging behaviors might be rich enough for further analyses. In order to predict users' personality based on enough digital records of micro-blogging behaviors, active users were selected as research objects in this study.

Sina Weibo officially defines active users as those who sign in Sina Weibo frequently. However, because of its unavailability (digital records of users' login history are not available for download through APIs), the official definition of active users might not be suitable for our study. In this study, we chose active users by considering both (a) total number of micro-blogs updates (TMU) and (b) average count of micro-blogs updates per day (AMUD).

**Figure 1:**
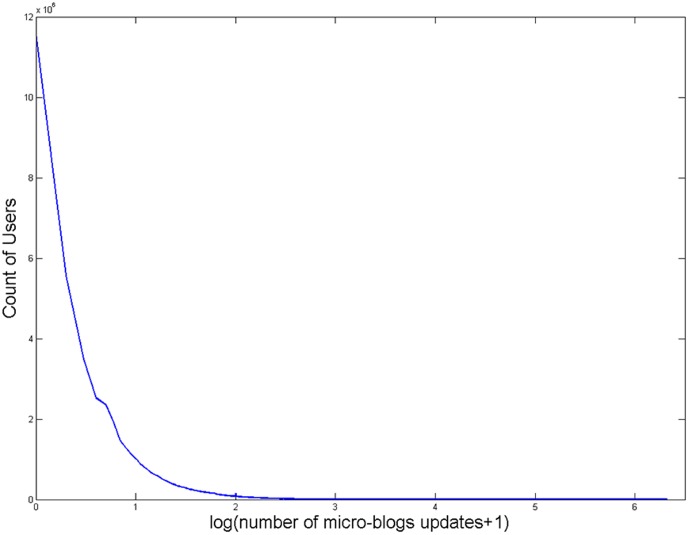
The Distribution of Users' Total Number of Micro-Blogs Updates (*n* = 99,925,821).

Specifically, because of Sina Weibo's data access control & user account management policy, we were not allowed to download detailed data of all 99,925,821 users. Instead, we downloaded brief information of 99,925,821 users on April 18, 2012, including number of followers, friends whom a user follows and micro-blogs updates. Types of users' brief information were downloaded since the initial registration. For 99,925,821 users, the distribution of TMU was shown in **Fig. 1** (mean  = 136.65±788.87), which followed an approximate Zipf distribution. Then, to filter out inactive users and remain enough active users for further analyses, we selected 6,047,966 users who had more than 532 micro-blogs updates (136.65+0.5×788.87). Among selected 6,047,966 users, we successfully downloaded detailed information of 5,919,087 users. Downloading detailed information of remaining 128,879 users was prohibited due to Sina Weibo's data access control & user account management policy.

**Figure 2:**
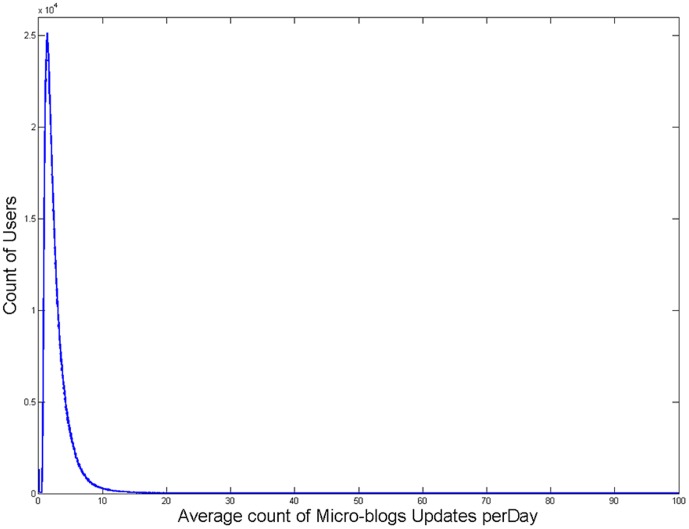
The Distribution of Users' Average Count of Micro-Blogs Updates per Day (*n* = 5,807,999).

Among 5,919,087 users with downloaded detailed information, the data from 111,088 users were excluded because they deleted their micro-blogs while we were downloading the detailed information. For the remaining 5,807,999 users, the distribution of AMUD was shown in **Fig. 2** (mean  = 2.84±2.57), which implied that most users post less than 10 micro-blogs per day. Based on the distribution of AMUD, users whose AMUD were between 2.84 and 40 would be selected for further analyses. Specifically, the lower boundary (2.84) implied the average count of AMUD and the upper boundary (40) served as a threshold for excluding extreme users. It is worthy to note that, among Sina Weibo users, there are a lot of extreme users, including VIP users (e.g., movie stars and sports stars) and advertisers. Because most of them update micro-blogs just for business purposes, it makes little sense to analyze their micro-blogging behaviors.

In summary, we selected active users by four criterions: (a) including user who had more than 532 micro-blogs after registration, (b) including user whose AMUD was between 2.84 and 40 after registration, (c) excluding user who had not published micro-blogs within last three months before this study and (d) excluding user whose last micro-blog was published within one month right after registration.

Finally, we selected a total of 1,953,485 active Sina Weibo users.

#### Collecting Data

**Figure 3:**
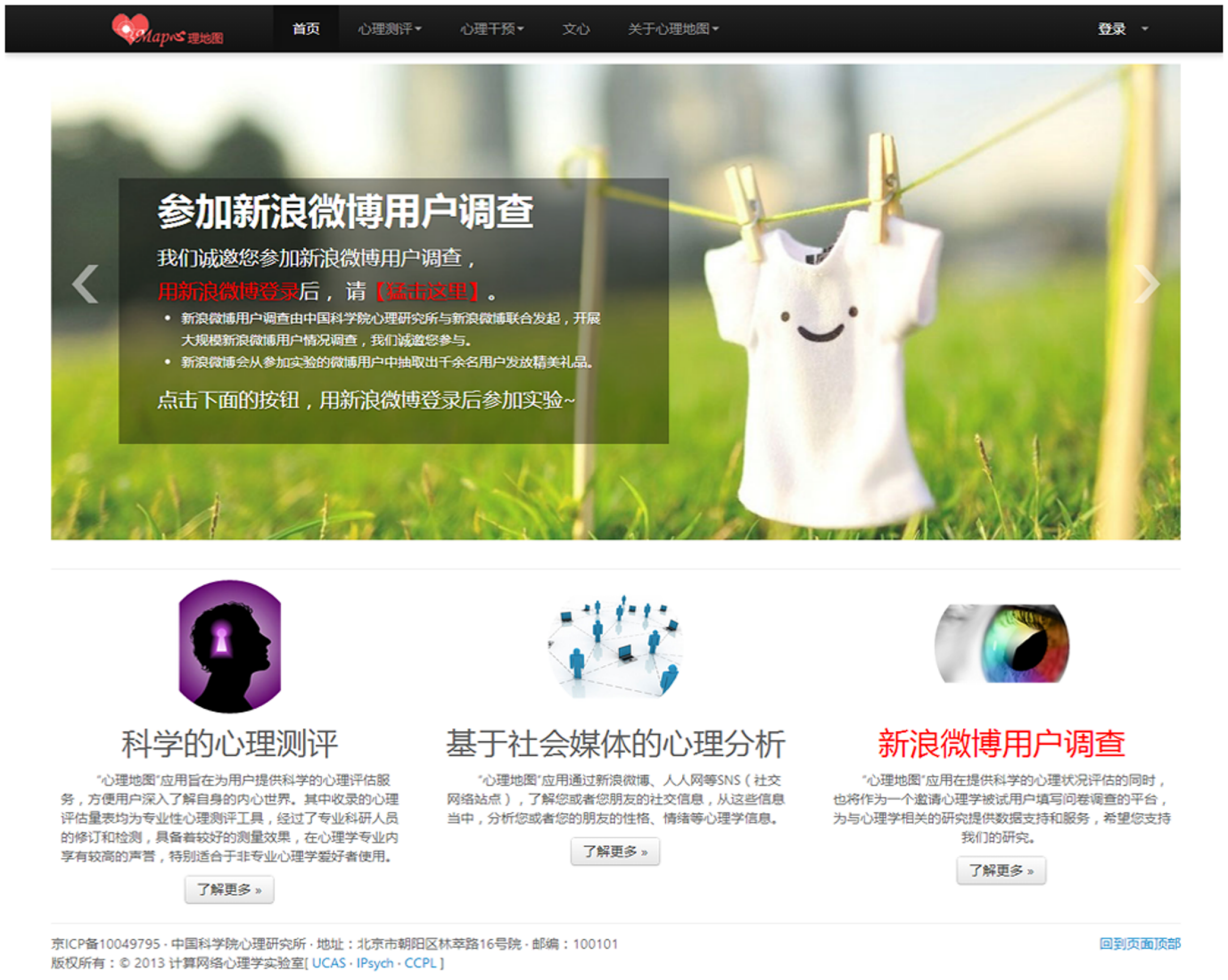
User Interface of a Weibo-Based Application Named “XinLiDiTu”.

In order to conduct online research and collect data, we build a Weibo-based application named “XinLiDiTu” (http://ccpl.psych.ac.cn:10002/) (see **Fig. 3**). Through “XinLiDiTu”, we could recruit participants, collect participants' scores on personality traits and digital records of micro-blogging behaviors, and finally pay recruiting fees.

In view of both web users' respond rate of Internet-based surveys (2% in experience) and our desired sample size (over 500 users), we randomly chose 30,000 users from 1,953,485 active users as our potential participants. We sent a participation invitation via Sina Weibo between May 30, 2012 and July 20, 2012. All potential participants were instructed to sign in to “XinLiDiTu” and presented with an electronic informed-consent form. Only those who agreed with the informed-consent form would be allowed to participate in this study. Then, we collected participants' scores on personality and downloaded digital records of their micro-blogging behaviors since the initial registration. After checking the quality of their work (providing valid answers on measures of personality traits and authoring “XinLiDiTu” to download digital records of their micro-blogging behaviors), we would pay them 30 Yuan (RMB) online by Internet banking within one week. Thus, this yielded 547 completed assessment.

**Figure 4:**
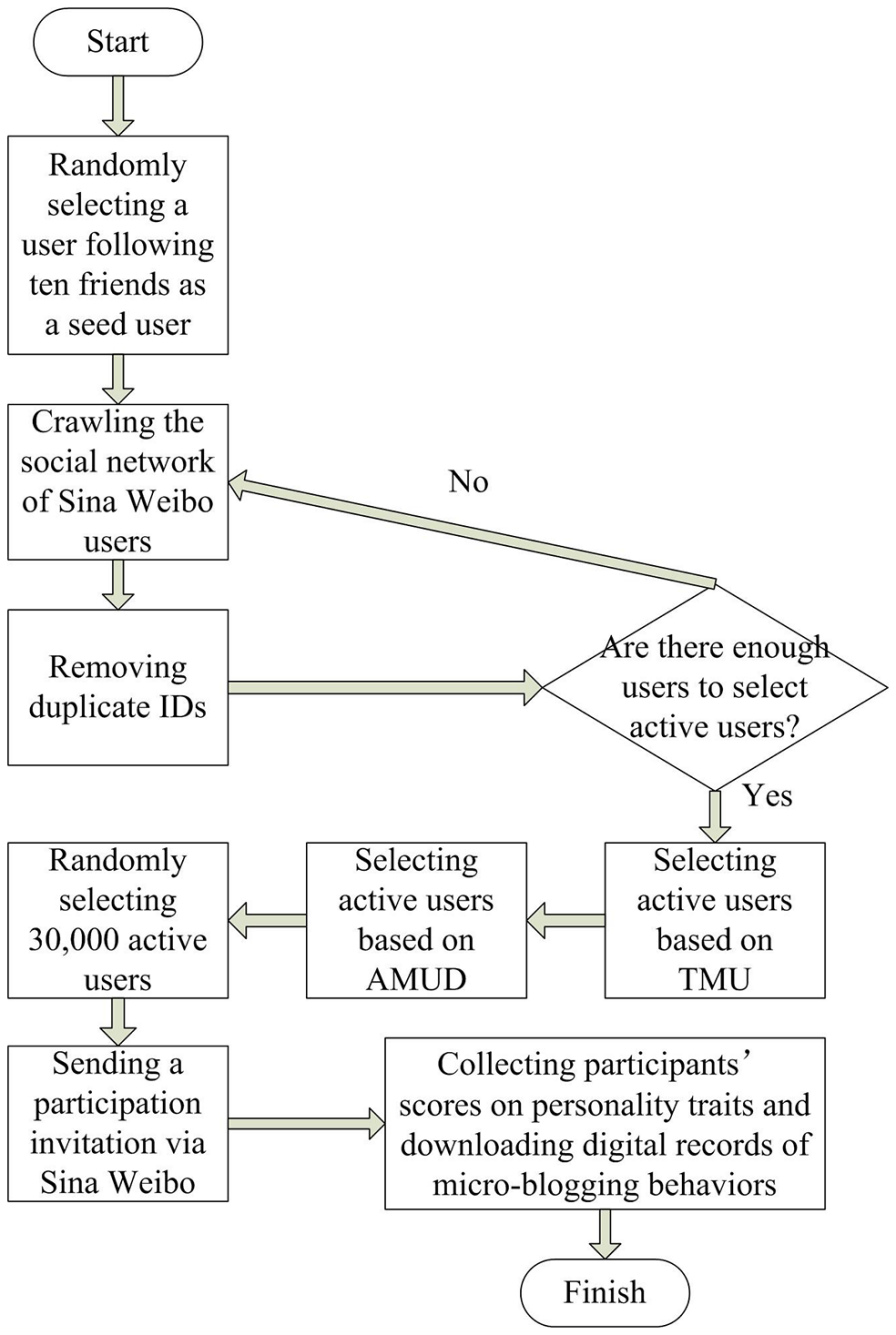
Procedure of this Study.

The whole procedure of this study was summarized in **Fig. 4**.

### Modeling Process

In this study, participants' behavioral features extracted from digital records of micro-blogging behaviors were treated as predictor variables and their scores on personality traits were treated as outcome variables.

First, we examined whether micro-blogging behaviors can be used to differentiate between users with high and low scores on each dimension of the Big Five Inventory. According to the Big-Five personality framework, one's personality is a hierarchical model with five bipolar personality dimensions (e.g., Extraversion versus Introversion). In BFI, one's personality profile is represented as continuous score. With an increasing difference in score between two respondents, it is easier to distinguish one from the other on certain personality dimension, which means that two respondents approximate to opposite ends of this personality dimension [5], [31]. Thus, in order to increase between-person variation, we focused on those participants with either high or low scores on each dimension of the Big Five Inventory. Because there was no official manual with published norms for both English and Chinese version of BFI, for each personality dimension, all participants were divided into one of three groups (i.e., high-scoring, middle-scoring and low-scoring group) based on thresholds (mean value ± standard deviation). Results of extreme grouping were shown in **Table 3**. Finally, we built classification models utilizing Support Vector Machine (SVM) methods [32], differentiating between participants with high and low scores on each dimension of the Big Five Inventory. These participants with middle scores on each dimension of the Big Five Inventory were not included in the process of SVM models training.

**Table 3:** Number of High-Scoring and Low-Scoring Participants on Each Dimension of the Big-Five Personality Traits (*n* = 547).

	Agreeableness	Conscientiousness	Extraversion	Neuroticism	Openness
High-Scoring Group	94	86	96	97	86
Low-Scoring Group	78	102	95	85	81

Second, in order to generalize the performance of measuring personality traits through micro-blogging behaviors over the full 547-sample size, we built regression models utilizing PaceRegression methods [33], predicting participants' scores on each dimension of the Big Five Inventory. That is, all high-scoring, middle-scoring and low-scoring participants on each personality dimension were included in the process of PaceRegression models training.

## Result

The process of building models was divided into four steps: (a) feature extraction, (b) feature selection, (c) model training and (d) model evaluation.

### Feature Extraction

We extracted two types of behavioral features (i.e., static features and dynamic features) from digital records of participants' micro-blogging behaviors.

#### Static Features

Static features refer to those features experiencing little changes over time, including four categories in this study: (a) profiles, (b) self-expression behaviors, (c) privacy settings and (d) interpersonal behaviors.

Specifically, (a) profiles included features like one's registration time and demographic information (e.g., gender). (b) Self-expression behaviors included features indicating the expression of one's personal image online (e.g., user's screen name, facial picture and self-statement on personal page). (c) Privacy settings included features indicating the protection of individual privacy (e.g., filtering out private messages and comments sent by strangers). (d) Interpersonal behaviors included features indicating the outcomes of social interaction between different users (e.g., number of friends whom a user follows, number of followers, categories of friends whom a user follows and categories of forwarded micro-blogs). Details of static features were shown in Appendix S1.

#### Dynamic Features

Dynamic features refer to those features experiencing obvious changes over time (e.g., per day). A dynamic feature could be represented as time series data. In this study, we extracted dynamic features following five steps: (a) listing initial dynamic features, (b) determining the length of observation time, (c) converting dynamic features into a two-dimensional matrix, whose rows represented hours and columns represented days, (d) exporting time series data from the two-dimensional matrix and (e) extracting final dynamic features from the time series data. In order to understand the process of dynamic features extraction in a better way, we took a kind of feature (the number of micro-blogs updates per hour) for example.

Specifically, (a) in this study, a total of 40 initial dynamic features were classified into four categories: micro-blogs updates (12), @ mentions (3), use of apps (6) and recordable browsing behaviors (19). Details of dynamic features were shown in Appendix S2.

**Figure 5:**
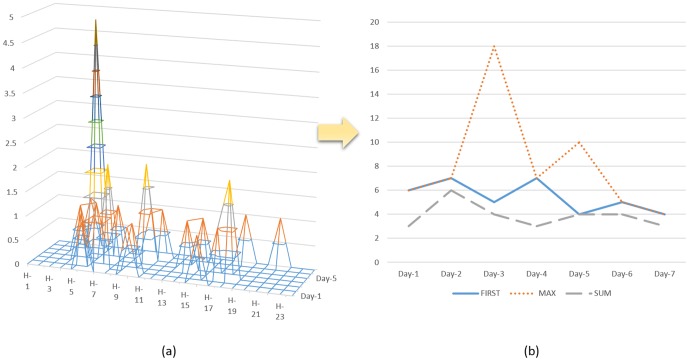
Exporting Time Series Data from Feature Matrix.

(b) For dynamic features extraction, determining the length of observation time is important. Different lengths of observation time would result in different kinds of dynamic features extraction, producing different optimal periods for predicting personality traits. In this study, different lengths of observation time were provided to select: 0-day, 7-day, 14-day, 21-day, 30-day, 37-day, 44-day, 51-day, 60-day, 67-day, 74-day, 81-day, 90-day, 97-day, 104-day, 111-day and 120-day. Among them, 0-day represented that there are no dynamic features involved in further analyses, while other periods represented that there are both static and dynamic features involved in further analyses.

(c) In this paper, we considered “hour” and “day” as two dimensions to describe temporal properties of dynamic features. The “hour” dimension indicated time difference in hours between 6:00 a.m. and the moment of target behavior occurring. The “day” dimension indicated time difference in days between the initiation of observation and the date of target behavior occurring. Then we can get a two-dimensional matrix. In this matrix, rows represented hours, columns represented days and elements represented counts of target behavior occurring in a certain time period. For example, if we would like to examine a user's micro-blogs updates in 7 days, we can get a 24 (hours) ×7 (days) matrix shown in **Fig. 5** **(a)**. An element in this matrix like {X_4, 12_ = 2} implied that a user updated two micro-blogs between 19:00 and 20:00 on the fourth day.

(d) Based on established two-dimensional matrix, we can export time series data (**Fig. 5** **(b)**). Taking micro-blogs updates as an example, we can export 5 behavior series form each user's feature matrix:

1) Row number indicating the first non-zero value in each column represented a time period (hours), when a user experienced an initial micro-blog update on each day.

2) Row number indicating the maximum value in each column represented a time period (hours), when user experienced an intensive micro-blogs updates on each day.

3) Counts of elements in each column represented a total number of micro-blogs updates on each day.

4) Counts of elements in each row represented a total number of micro-blogs updates in each hour across all 7 days.

5) Counts of all 24×7 elements represented a total number of micro-blogs updates on all 7 days.

(e) Finally, we extracted dynamic features from time series data. In this study, we extracted four kinds of features: mean value, variance, sum and weighted sum of behavior series.

In summary, we suggested 45 static features and 40 types of matrices. We exported 5 behavior series from each type of matrix and got a total of 200 behavior series (40×5). Then, we extracted four kinds of features from each behavior series and got a total of 800 dynamic features (40×5×4). Finally, we got a total of 845 features (45+800), including 45 static features and 800 dynamic features.

### Feature Selection

In order to maximize the value of adjusted R-square in modeling, we selected features from a total of 845 features utilizing StepWise methods [34]. For different personality dimensions, different types of features were selected. **Table 4** showed the number and performance of selected features in PaceRegression models. It is worthy to note that, for each model, the performance of selected features presented in **Table 4** was the optimal one for different lengths of observation time.

**Table 4:** Performance of Selected Features in PaceRegression Models (*n* = 547).

	Optimal Prediction Period	Number of Selected Features	Adjusted R-Square
Agreeableness	74-day	31	0.22
Conscientiousness	97-day	35	0.29
Extraversion	51-day	25	0.26
Neuroticism	74-day	30	0.26
Openness	74-day	26	0.23

### Model Training

For each observation period (17 observation periods in total), we trained SVM models and PaceRegression models based on methods of 5-fold cross-validation. Specifically, we randomly divided data into 5 subgroups with equal size. Then, each subgroup would be used to test the model built on other 4 subgroups. After 5 rounds of model training, we integrated 5 results of model training into a final model. It is worthy to note that, based on out-of-sample estimation method, during a process of cross-validation, testing data could not be included in training data. Thus, the generalization ability of models tested by 5-fold cross-validation would be ideal.

Besides, we used a toolkit called LibSVM (http://140.112.30.28/~cjlin) to build SVM models. The components of SVM model include a kernel function and its corresponding parameters. For RBF kernel function used in this study, both Gamma (g, a parameter in the RBF kernel function) and Cost (c, a balance between the accuracy and the generalization ability of model) are significant parameters determining the performance of a SVM model. To improve the performance of SVM models, in this study, such two parameters were tuned utilizing grid search method [35].

### Model Evaluation

Both established SVM models and PaceRegression models need to be evaluated by comparing their performance against corresponding baseline models. For SVM models, the baseline models were random models, which assigned labels of either high-scoring or low-scoring on each personality dimension to any participant randomly. For PaceRegression model, the baseline models were average models, which predicted individual personality profile based on the mean value of all 547 participants' scores on each personality dimension.

Specifically, we used accuracy estimation to evaluate the performance of SVM models and used Pearson correlation coefficient (CC) and relative absolute error (RAE) to evaluate the performance of PaceRegression models. For SVM models, the value of accuracy estimation represented the percentage of cases correctly predicted. The expected accuracy of a baseline model was 50%. For PaceRegression Models, the CC represented correlations between predicted scores and actual scores, which implied the degree of consistency between both of them. Besides, RAE presented a ratio of mean absolute error (MAE) between established model and baseline model. The expected CC of a baseline model was 0 and the expected RAE of a baseline model was 100%.

**Figure 6:**
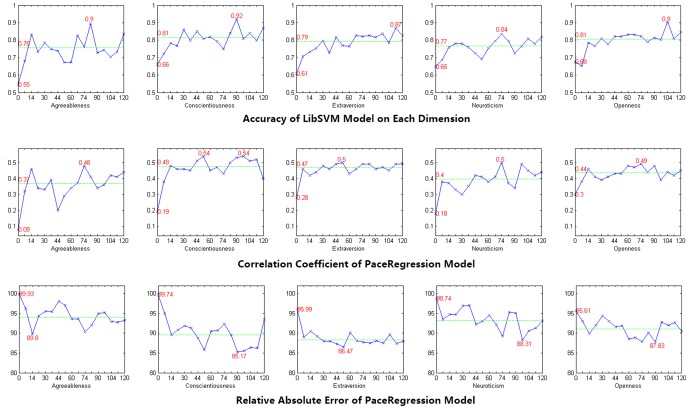
Results of Model Evaluation.

Results of model evaluation were shown in **Fig. 6**, which indicated changes in values of evaluation parameters (e.g., accuracy estimation, CC and RAE) on each personality dimension for different lengths of observation time. In **Fig. 6**, the green lines represented the average estimation of evaluation results of all 8 non-zero-day models.

For each model, the evaluation results of any non-zero-day were better than the evaluation results of 0-day. Then, after including dynamic features, models on different personality dimensions performed differently in the growth of performance evaluation across different lengths of observation time. For example, the model of Agreeableness improved rapidly, while the model of Openness improved slowly.

Besides, counts of behavioral features selected for predicting personality dimensions were inconsistent between SVM models and PaceRegression Models. Specifically, for SVM models, the number of behavioral features selected in models for the Extraversion, Agreeableness, Conscientiousness, Neuroticism and Openness were 27, 39, 27, 36 and 42 respectively. While, for PaceRegression models, the number of behavioral features selected in models for the Extraversion, Agreeableness, Conscientiousness, Neuroticism and Openness were 25, 31, 35, 30 and 26 respectively. Those behavioral features selected simultaneously in both SVM and PaceRegression models were regarded as important features for predicting each personality dimension. Because SVM coefficients of important features could not indicate linear relationships between personality traits and micro-blogging behaviors, PaceRegression coefficients of important features were shown in **Table 5**.

**Table 5:** PaceRegression Coefficients of Important Features for Predicting Personality Dimensions (*n* = 547).

Extraversion	Agreeableness	Conscientiousness	Neuroticism	Openness
Feature 1	Feature 5	Feature 10	Feature 13	Feature 1
(β = 0.7551)	(β = −0.2434)	(β = 0.9968)	(β = −1.1922)	(β = 1.0274)
Feature 2	Feature 6	Feature 11	Feature 14	Feature 19
(β = 0.6704)	(β = −0.4580)	(β = −0.6978)	(β = −0.6246)	(β = 0.0007)
Feature 3	Feature 7	Feature 12	Feature 5	Feature 20
(β = 0.0610)	(β = 0.1997)	(β = −0.0075)	(β = 0.3756)	(β = 0.1189)
Feature 4	Feature 8		Feature 15	Feature 21
(β = 0.6846)	(β = −0.0469)		(β = 0.5821)	(β = 0.3794)
	Feature 9		Feature 16	
	(β = 0.2432)		(β = −0.1727)	
			Feature 17	
			(β = −1.4693)	
			Feature 18	
			(β = 1.2599)	

Notes. Feature 1 =  Having certified users in mutual friends (yes  = 1, no  = 0). Feature 2 =  Number of friends whom a user follows. Feature 3 =  Numerical order of the date in observation period for updating positive emoticons the most. Feature 4 =  Standard deviation of hours (1–24, ranging from 6:00 a.m. to the next 6:00 a.m.) in every 24-hour period for forwarding the first micro-blog whose total number of comments and forwards have been over 5. Feature 5 =  The hour (1–24, ranging from 6:00 a.m. to the next 6:00 a.m.) in a 24-hour period for usually sending @ mentions to friends whom a user follows the most. Feature 6 =  The day of the week (1–7, ranging from Monday to Sunday) for usually forwarding micro-blogs updated by friends whom a user follows the most. Feature 7 =  Standard deviation of hours (1–24, ranging from 6:00 a.m. to the next 6:00 a.m.) in every 24-hour period for forwarding micro-blogs updated by friends whom a user follows the most. Feature 8 =  Numerical order of the date in observation period for forwarding micro-blogs updated by apps for information purpose the most. Feature 9 =  The hour (1–24, ranging from 6:00 a.m. to the next 6:00 a.m.) in a 24-hour period for usually forwarding micro-blogs updated by accounts of organization the most. Feature 10 =  Having a register account of Sina Blogging as well (yes  = 1, no  = 0). Feature 11 =  Standard deviation of numbers in updating negative emoticons every day. Feature 12 =  Summation of hours (1–24, ranging from 6:00 a.m. to the next 6:00 a.m.) in every 24-hour period for updating the first micro-log attached with @ mentions. Feature 13 =  Use of the first personal pronoun subjects in creating self-statement (yes  = 1, no  = 0). Feature 14 =  Number of user's interested trending topics shared by over 10000 users. Feature 15 =  The day of the week (1–7, ranging from Monday to Sunday) for usually updating emoticons the most. Feature 16 =  The hour (1–24, ranging from 6:00 a.m. to the next 6:00 a.m.) in a 24-hour period for usually forwarding micro-blogs updated by apps for business purpose the most. Feature 17 =  Standard deviation of numbers in forwarding micro-blogs updated by accounts of website every day. Feature 18 =  The hour (1–24, ranging from 6:00 a.m. to the next 6:00 a.m.) in a 24-hour period for usually updating original micro-blogs with maximum content length. Feature 19 =  Number of favorite micro-blogs which user collects. Feature 20 =  The hour (1–24, ranging from 6:00 a.m. to the next 6:00 a.m.) in a 24-hour period for usually using apps for business purpose the most. Feature 21 =  The hour (1–24, ranging from 6:00 a.m. to the next 6:00 a.m.) in a 24-hour period for usually updating positive emoticons the most.

## Discussion

This study explored the association between active users' digital records of micro-blogging behaviors and their personality traits. We downloaded active users' digital records of micro-blogging behaviors utilizing APIs of Sina Weibo and collected scores on personality traits through online survey. Finally, we built models for predicting personality traits based on active users' micro-blogging behaviors.

### Modeling Performance

From the perspective of prediction accuracy, for SVM models, as shown in **Fig. 6**, values of accuracy estimation were over 84% on each personality dimension, which were obvious higher than the accuracy estimation of baseline model (50%). This suggested that micro-blogging behaviors can be used to identify users with either high-scoring or low-scoring on each personality dimension efficiently. Then, for PaceRegression models, the CC ranged from 0.48 to 0.54, which implied moderate correlations between predicted scores and actual scores. Besides, the RAE ranged from 85.17% to 89.8%. Results indicated that, compared with baseline models, the performance of established models were satisfying. This suggested that micro-blogging behaviors can be used to predict active users' scores on each personality dimension precisely.

From the perspective of interpretability issues in modeling, according to **Table 5**, the relationships between personality dimensions and micro-blogging behaviors could be confirmed by theories of the Big-Five personality framework. (a) For dimension of Extraversion, individuals who are high in Extraversion tend to experience positive emotional states and have broad social communications with others. They are described as sociable, gregarious, assertive, talkative and active. In this study, micro-blogging users who scored high on Extraversion built extensive and strong connections with other micro-blogging users (Feature 1 and Feature 2), which implied they would get social benefit from the Internet-mediated communication. Besides, they also had an increasing propensity of positive emotion experience during observation period (Feature 3) and got used to communicating with online friends at any time (Feature 4). (b) For dimension of Agreeableness, individuals who are high in Agreeableness tend to get along well with others. They are described as courteous, flexible, trusting, good-natured, cooperative, forgiving, soft-hearted and tolerant. In this study, micro-blogging users who scored high on Agreeableness managed to avoid communicating with online friends at undesirable or private time (Feature 5 and Feature 6). (c) For dimension of Conscientiousness, individuals who are high in Conscientiousness tend to have the tendency of powerful impulse control and persistent goal pursuit. They are described as careful, thorough, responsible, organized, well-planned, hardworking, achievement-oriented and persevering. In this study, micro-blogging users who scored high on Conscientiousness would like to communicate with online friends regularly (Feature 11) and earnestly (Feature 10 and Feature 12) as well. (d) For Neuroticism, individuals who are high in Neuroticism tend to experience negative emotional states and perceive oneself and the world negatively. They are described as anxious, depressed, angry, embarrassed, emotional, worried and insecure. In this study, micro-blogging users who scored high on Neuroticism might have low level of self-identification (Feature 13). They could not help communicating with online friends at any time they want (Feature 5 and Feature 18) and seemed to avoid following trending topics which might lead to emotional arousal (Feature 14). (e) For Openness, individuals who are high in Openness tend to have broad interests and are willing to experience something unusual. They are described as imaginative, cultured, curious, original, broad-minded, intelligent and artistically sensitive. In this study, micro-blogging users who scored high on Openness had collected a number of favorite micro-blogs (Feature 19), which implied they had a wide range of interest online.

### Length of the Observation Time

Blackman found that an increasing length of the observation time would improve modeling performance for predicting personality traits [36]. This conclusion was consistent with our findings. **Fig. 6** showed that, with an increasing length of the observation time, modeling performance could be improved. Besides, each model had its own optimal observation period for predicting scores on certain personality dimension. For example, the optimal observation period for predicting scores on dimension of Extraversion, Agreeableness, Conscientiousness, Neuroticism and Openness ranged from 74 days to 111 days.

### Differences in Modeling Performance across Personality Dimensions

According to **Fig. 6**, there were differences in modeling performance across personality dimensions. We found that modeling performance in predicting Openness dimension improved rapidly and experienced convergence after 30 days, while modeling performance in predicting other dimensions were likely to rise slowly. That is to say, because of its good validity and stability over time, Openness dimension could be predicted through micro-blogging behavior easily, which was consistent with previous studies [37]. Conversely, for Agreeableness dimension, the modeling performance was unstable over time. It suggested that, compared with other dimensions, it could be harder to predict Agreeableness dimension through micro-blogging behaviors.

Limitations existed in this study. (a) Limited length of the observation time needs to be extended. In this study, the maximum observation time was 120 days. We were not sure about whether the collection of micro-blogging behaviors in a longer observation period would further improve modeling performance. Moreover, length of the observation time in this study might not be adequate enough for detecting the pattern of active users' micro-blogging behaviors completely. (b) In this study, in order to improve models fit, we selected features based on criterion of adjusted R-square, which might not be sensitive enough to predict personality traits. Innovative methods for improving the performance of features selection need to be discovered. (c) This study only focused on micro-blogging behaviors and neglected to analysis text contents of micro-blogs. The integration of behavior analysis and text analysis would improve modeling accuracy for predicting personality traits. (d) In order to control the disturbing effect of the experience of using micro-blogging on modeling, it would be better to select participants based on their registration time.

In this paper, we demonstrated that micro-blogging behaviors can be used to predict active users' personality traits with different optimal observation periods. This work provides a basis for improving the traditional method of psychological testing (e.g., questionnaire survey). Specifically, the deliberative and time-consuming process of collecting self-report data is conducted in an intrusive manner under experimental conditions, while the automatic and momentary process of collecting micro-blogging data is conducted in a non-intrusive manner under ecological conditions. With the help of computational models built on actual web use behaviors, the process of measuring psychological features can be automatic, momentary and ecological. It implies the establishment of a ubiquitous web-based psychological laboratory, which will be beneficial to the public in the future from both research (e.g., enabling retrospective and longitudinal research to address issues of how psychological features change over time and across contexts) and practical perspectives (e.g., delivering digital personnel assessment, personalized recommendation online and population-based detection of mental health problems).

## Supporting Information

Appendix S1 **Details of Static Features.**
(DOCX)

Appendix S2 **Details of Dynamic Features.**
(DOCX)
