# Correction: Predicting Active Users' Personality Based on Micro-Blogging Behaviors

**DOI:** 10.1371/journal.pone.0098489

**Published:** 2014-05-19

**Authors:** The PLOS ONE Staff

The number of static features is incorrect in several locations in the manuscript. The correct number of static features is 39. As a result, several calculations in the manuscript are incorrect.

The fifth sentence of the abstract should read:

“After extracting 839 micro-blogging behavioral features, we first trained classification models utilizing Support Vector Machine (SVM), differentiating participants with high and low scores on each dimension of the Big Five Inventory.”

The final paragraph in the “Dynamic Features” Section should read:

“In summary, we suggested 39 static features and 40 types of matrices. We exported 5 behavior series from each type of matrix and got a total of 200 behavior series (40 × 5). Then, we extracted four kinds of features from each behavior series and got a total of 800 dynamic features (40 × 5 × 4). Finally, we got a total of 839 features (39 + 800), including 39 static features and 800 dynamic features.”

The first sentence in the “Feature Selection” Section should read:

“In order to maximize the value of adjusted R-square in modeling, we selected features from a total of 839 features utilizing StepWise methods [34].”

In the Supporting Information Files, Appendix S1 is incorrectly labelled as Appendix S2, and Appendix S2 is incorrectly labelled as Appendix S1.
