# Supplementary material for: Predicting Active Users' Personality Based on Micro-Blogging Behaviors
Source: PLoS One. 2014 Jan 22;9(1):e84997. doi: 10.1371/journal.pone.0084997 (PMC3898945; doi:10.1371/journal.pone.0084997)
Supplement: Appendix S1 — Details of Static Features. (DOCX) [file pone.0084997.s001.docx]

**Appendix S2.** Details of Dynamic Features

| **Categories** | **Contents** |
| --- | --- |
| Micro-Blogs Updates | updating micro-blogs |
|  | updating micro-blogs attached with emoticons |
|  | updating micro-blogs attached with positive emoticons |
|  | updating micro-blogs attached with negative emoticons |
|  | delivering micro-blogs via desktop computer |
|  | delivering micro-blogs via other equipment |
|  | length of original micro-blogs |
|  | length of original micro-blogs whose duplicate words have been deleted |
|  | updating micro-blogs whose total number of comments and forwards have been over 5 |
|  | updating original micro-blogs attached with emoticons |
|  | updating original micro-blogs attached with positive emoticons |
|  | updating original micro-blogs attached with negative emoticons |
|  |  |
| @ Mentions | number of friends targeted by @ mentions |
|  | number of @ mentions in original micro-blogs updates |
|  | number of friends targeted by @ mentions in original micro-blogs updates |
|  |  |
| Use of Apps | use of any apps |
|  | use of apps for information purpose |
|  | use of apps for business purpose |
|  | use of apps for communication purpose |
|  | use of apps for entertainment purpose |
|  | use of apps for other purpose |
|  |  |
| Recordable Browsing Behaviors | forwarding micro-blogs |
|  | forwarding micro-blogs updated by apps |
|  | forwarding micro-blogs updated by apps for information purpose |
|  | forwarding micro-blogs updated by apps for business purpose |
|  | forwarding micro-blogs updated by apps for communication purpose |
|  | forwarding micro-blogs updated by apps for entertainment purpose |
|  | forwarding micro-blogs updated by apps for other purpose |
|  | forwarding micro-blogs updated by certified personal users |
|  | forwarding micro-blogs updated by accounts of government |
|  | forwarding micro-blogs updated by accounts of corporation |
|  | forwarding micro-blogs updated by accounts of media |
|  | forwarding micro-blogs updated by accounts of campus |
|  | forwarding micro-blogs updated by accounts of website |
|  | forwarding micro-blogs updated by accounts of app |
|  | forwarding micro-blogs updated by accounts of organization |
|  | forwarding micro-blogs updated by personal users who are honored as Weibo Girl |
|  | forwarding micro-blogs updated by personal users who are honored as Junior Expert |
|  | forwarding micro-blogs updated by personal users who are honored as Senior Expert |
|  | forwarding micro-blogs whose total number of comments and forwards have been over 5 |
