# Supplementary material for: Predicting Active Users' Personality Based on Micro-Blogging Behaviors
Source: PLoS One. 2014 Jan 22;9(1):e84997. doi: 10.1371/journal.pone.0084997 (PMC3898945; doi:10.1371/journal.pone.0084997)
Supplement: Appendix S2 — Details of Dynamic Features. (DOCX) [file pone.0084997.s002.docx]

**Appendix S1.** Details of Static Features

| **Categories** | **Contents** |
| --- | --- |
| Profile | gender |
|  | city code |
|  | province code |
|  | length of registration |
|  |  |
| Self-presentation | use of default avatar (or not) |
|  | length of self-statement |
|  | use of the first personal pronoun subjects in creating self-statement (or not) |
|  | content length of personalized domain name |
|  | use of numeric characters in creating personalized domain name (or not) |
|  | being a certified user of Sina Weibo (or not) |
|  | types of certification acquired |
|  | length of a request for getting certification |
|  | length of screen name |
|  | number percentage of Chinese characters in screen name |
|  | owning a register account of Sina Blogging |
|  | duplication of a domain name between Sina Weibo and Sina Blogging (or not) |
|  | number of a user’s personal tags used by 0-100 users |
|  | number of a user’s personal tags used by 100-10000 users |
|  | number of a user’s personal tags used by over 10000 users |
|  | number of a user’s interested trending topics shared by 0-100 users |
|  | number of a user’s interested trending topics shared by 100-10000 users |
|  | number of a user’s interested trending topics shared by over 10000 users |
|  |  |
| Security Settings | authorization for receiving private messages from any user of Sina Weibo (or not) |
|  | authorization for receiving comments from any user of Sina Weibo (or not) |
|  | authorization for attaching geographic information to updated micro-blogs (or not) |
|  |  |
| Social Communications | number of micro-blogs updates |
|  | number of friends whom a user follows |
|  | number of followers |
|  | number of mutual friends |
|  | number percentage of original micro-blogs in micro-blogs updates |
|  | number of micro-blogs which a user collects |
|  | number percentage of mutual friends in followers |
|  | number percentage of mutual friends in friends whom a user follows |
|  | having certified users in mutual friends (or not) |
|  | types of user objects whose micro-blogs have been forwarded by a user |
|  | the type of a user object whose micro-blogs have been forwarded the most by a user |
|  | the type of a user object whose micro-blogs have been forwarded the second most by a user |
|  | the type of a user object whose micro-blogs have been forwarded the third most by a user |
|  | having certified users in friends whom a user follows (or not) |
